# New York City jails: COVID discharge policy, data transparency, and reform

**DOI:** 10.1371/journal.pone.0262255

**Published:** 2022-01-19

**Authors:** Eli Miller, Bryan D. Martin, Chad M. Topaz

**Affiliations:** 1 Department of Mathematics and Statistics, Williams College, Williamstown, MA, United States of America; 2 Department of Statistics, University of Washington, Seattle, WA, United States of America; 3 Institute for the Quantitative Study of Inclusion, Diversity, and Equity, Williamstown, MA, United States of America; University of Maryland at College Park, UNITED STATES

## Abstract

During the early stages of the COVID-19 pandemic in 2020, Mayor Bill de Blasio ordered the release of individuals incarcerated in New York City jails who were at high risk of contracting the disease and at low risk of committing criminal reoffense. Using public information, we construct and analyze a database of nearly 350,000 incarceration episodes in the city jail system from 2014—2020, paying special attention to what happened during the week of March 23—29, 2020, immediately following the mayor’s order. In concordance with de Blasio’s stated policy, we find that being discharged during this focus week is associated with a lower probability of readmission as compared to being discharged during the same calendar week in previous years. Furthermore, comparing the individuals discharged during the focus week of 2020 to those discharged during the same calendar week in previous years, we find that the former group was, on average, slightly older than the latter group, although the difference is not large. Additionally, the individuals in the former group had spent substantially longer in jail than those in the latter group. With the release of long-serving individuals demonstrated to be feasible, we also examine how the jail population would have looked over the past six years had caps in incarceration been in place. With a cap of one year, the system would experience a 15% decrease in incarceration. With a cap of 100 days, the reduction would be over 50%. Because our results are only as accurate as New York City’s public-facing jail data, we discuss numerous challenges with this data and suggest improvements related to the incarcerated individual’s age, gender, race, and more. Finally, we discuss the policy implications of our work, highlight some opportunities and challenges posed by incarceration caps, and suggest key areas for reform. One such reform might involve identifying and discharging low-risk individuals sooner in general, which might be feasible given the de Blasio administration’s actions during the early stages of COVID-19.

## Introduction

New York City’s COVID-19 outbreak surged in late March 2020 and had immediate impact on the city’s jail populations. As of Saturday, March 14, Rikers Island, the largest of the city’s jails, had confirmed 38 COVID-19 cases, up from eight the previous day. Under the simple assumption of exponential growth early in a pandemic, the entire jail system, with approximately 5,300 incarcerated individuals and 1,000 employees, could have been infected in two weeks. Jails had already started taking public health precautions such as limiting visitors, equipping guards with personal protective equipment, cleaning shared surfaces more frequently, and mandating that individuals in bunk beds sleep head-to-toe. Many jail staffers worried that these measures would not be sufficient to prevent a mass outbreak [1].

City officials and public defenders called attention to vulnerable populations within the city jail system. For example, the chief medical officer of the city’s Correctional Health Services tweeted, “We cannot socially distance dozens of elderly men living in a dorm, sharing a bathroom” [2]. Another physician within the jail system stated, “The only meaningful public health intervention here is to depopulate the jails dramatically” [1].

On March 24, Mayor Bill de Blasio ordered the immediate discharge of individuals who were at high risk of contracting COVID-19 and low risk of reoffending [3]. Public defenders urged de Blasio to widen his criteria, highlighting conditions inside the jails, where individuals were frequently denied basic sanitary products or protective equipment [4]. De Blasio’s administration responded, and by mid-April, as we will show, the jail population had decreased by 30%.

Whom did the city decide was both at a high risk of contracting the virus and a low risk of reoffending? What do these decisions imply about potential future reforms to the city’s incarceration policy? Our study answers these questions by studying public data from the New York City jail system over the past six years.

### Jails and criminal justice in New York City

In the United States, prisons incarcerate individuals who have committed serious crimes and have long sentences. In contrast, jails play multiple roles in the justice system. The primary purpose of a jail is to provide temporary detainment to pre-trial defendants, either to ensure their attendance at trial or to mitigate the immediate threat they pose to public safety. But depending on the municipality, a jail may also contain locally sentenced individuals serving short sentences, state-sentenced individuals awaiting transfer to a long-term state prison, parole or probation violators awaiting a hearing, or detainees of federal law enforcement agencies [5]. As a result, while US prisons are usually segregated by gender, age, and offense level, a US jail holds a widely diverse population in all three categories. Researchers have long acknowledged the role of jail population growth in the meteoric rise of the national incarceration rate over the last 40 years [6]. During this time, the heterogeneous nature of the country’s decentralized, municipal jail system has made jail populations difficult to analyze, manage, and reform [7].

In 2015, the need for sweeping reform to, specifically, the jail system of New York City became more stark in the wake of Kalief Browder’s suicide. Browder, then sixteen years old, had been arrested for allegedly stealing a backpack in 2010, detained for three years in New York City’s Rikers Island jail awaiting a trial date, and then released due to a lack of evidence. Due to the persistent abuse and violent treatment that he received from correctional officers, Browder’s mental health had quickly deteriorated, and during his three years in jail, he had committed multiple acts of self-harm, including at least one suicide attempt [8]. “Being home is way better than being in jail,” Browder told a journalist from *The New Yorker*, a year after his release, and six months before his suicide. “But in my mind right now I feel like I’m still in jail, because I’m still feeling the side effects from what happened in there…I feel like I was robbed of my happiness” [9].

Browder’s story, as well as hundreds of other accounts of systemic abuse and injustice in the Rikers Island jail [10, 11], inspired criminal justice advocates to fight for change. In October 2019, the city council approved an $8 billion plan to close Rikers and replace it with new, smaller facilities scattered throughout the city [12]. These facilities would have a combined capacity of 3,300, which would require a reduction in jail population of over 50%. Thus, by necessity, jail population reduction became a priority for the de Blasio administration.

To accomplish this goal, jail reformers urged the state to enact a longtime policy objective: bail reform. The United States justice system’s reliance on pretrial bail has been widely criticized as a mechanism of jailing people simply for being poor. It has also come under focus as a driver of racial inequity, because a disproportionate number of defendants in New York City who cannot pay bail are Black or Hispanic [13]. In July 2018, New York City’s Independent Commission on Criminal Justice and Incarceration Reform wrote that “Money bail is the preeminent driver of the jail population in New York City.” As of May 2018, nearly 75% of individuals in jail were pretrial, with the vast majority there simply because they could not afford bail [14]. The Commission found that an expanded supervised release program, under which defendants would be allowed to remain in the community as long as they submitted to frequent monitoring by city-employed social workers, would lower jail populations while successfully ensuring that defendants attended their court dates.

New York State helped expedite this reform in March, 2019 by abolishing the use of cash bail for all defendants, save those charged with certain violent felonies. This bail reform law went into effect January 1, 2020, but judges began applying it retroactively to individuals already in jail for not posting bail in November, 2019 [15]. As a result, the city’s jail population dropped from approximately 7,000 at the beginning of November to just over 5,300 at the beginning of February, 2020 ––– a reduction of nearly 25%. Three months later, however, Governor Andrew Cuomo slightly rolled back the reform by allowing bail to be used for defendants charged with an additional list of 25 offenses [16].

### Jails and infectious disease

As compared to the United States’ general population, the incarcerated population experiences higher disease contagion because they are in a congregate setting, and because there is an over-representation of individuals from racial and socioeconomic groups vulnerable to poor health outcomes [17]. The spread of illness in jails, specifically, is of interest to public health researchers because it has significant influence on spread in the surrounding communities, as most individuals in jails are discharged within a few weeks [18]. In particular, people incarcerated across the country [19] and in New York City [12] have contracted COVID-19 at a dramatically higher rate than the general population. Reducing jail populations has had a demonstrable effect on reducing spread of COVID-19 both within jails [20, 21] and in the community [22].

### Our study

Given the ongoing debates about criminal justice reform, and given the continuing worldwide struggle to find effective public policies to curtail the spread of COVID-19, the study of New York City’s jail discharge policy in March 2020 is a pressing matter. The rest of this paper is organized as follows.

In Methods, we present our procedure for gathering, reconciling, and cleaning data that we obtain from the city’s Open Data Portal. Our data set consists of nearly 350,000 records, each of which describes an incarceration episode.

In Analysis and Results, we report our findings. The city jail population did decrease substantially from March 23—29, 2020, following de Blasio’s policy charge. We find that being discharged during this focus week is associated with a lower probability of readmission as compared to the same calendar week in previous years. Furthermore, comparing the individuals discharged during the focus week of 2020 to those discharged during the same calendar week in previous years, we find that the former group was, on average, slightly older than the latter group, although the difference is not large. Additionally, the individuals in the former group had spent substantially longer in jail than those in the latter group.

With the release of long-serving individuals demonstrated to be feasible, and keeping in mind that the jail system is intended for short-term incarceration, in Policy Simulation, we examine how the jail population would have looked over the past six years had caps in incarceration been in place. With a cap of one year, the system would experience a 15% decrease in incarceration. With a cap of 100 days, the reduction would be over 50%.

Because our results are only as accurate as New York City’s public-facing jail data, in Data Recommendations, we discuss numerous challenges with this data and suggest improvements. These improvements would address issues related to the incarcerated individual’s age, gender, race, and more.

Finally, in Discussion and Conclusions, we discuss policy implications of our work, highlight some opportunities and challenges posed by incarceration caps, and suggest key areas for reform. One such reform might involve identifying and discharging low-risk individuals sooner in general, which might be feasible given the de Blasio administration’s actions during the early stages of COVID-19.

## Methods

We obtain data from the New York City Open Data Portal [23], which was built for compliance with the city’s Open Data Law, signed by Mayor Michael Bloomberg in 2012. The portal includes two data sets entitled *Inmate Admissions* and *Inmate Discharges*. These data sets provide historical information and are the basis of our study. Additionally, the portal includes a data set entitled *Daily Inmates in Custody*, which provides information about individuals who are currently incarcerated. This data set is not cumulative in time, is replaced online every day, and is not archived, rendering it unusable for historical study. However, we use it to resolve a small number of data anomalies, as we describe later.

In the following subsections, we detail the production of our final data set. The primary steps in this process are data acquisition, reconciliation of erroneous records, and cleaning of demographic and other variables, including removal of outliers.

### Data acquisition

The *Inmate Admissions* data set (ADS) and *Inmate Discharges* data set (DDS) files are updated at the start of each month. We downloaded both in.csv format on January 9, 2020, after their January 1 update. Upon download, ADS had 335,491 records and DDS had 339,910 records. Both files cover the time period 2014 through 2020, and both contain the following fields:

- ID number, a unique identifier for each incarcerated individual,
- date and time of admission into jail system,
- date and time of discharge from jail system,
- race
- gender
- a code describing the individual’s status within the justice system, and
- a code corresponding to New York state law that specifies the most significant charge associated with the incarceration.

Additionally, DDS provides the age of each individual at discharge.

### Data reconciliation

ADS and DDS provide similar information and have identical columns, with the exception that DDS includes age at discharge time. Crucially, though, the two files are not identical. For example, any individuals admitted prior to January 1, 2014 but discharged afterwards would appear in DDS but not in ADS. Conversely, any individuals who had been admitted but were not discharged prior to January 1, 2020 would appear in ADS but not in DDS. Still, beyond these explicable discrepancies, we find: records that are present in one but not both files; records that are in both files but with some fields missing data in one or both files; and records that are in both files but with conflicting information. Additionally, within each data set, we find records that are near-duplicates but that disagree on a subset of variables. We recognize four categories of records that must be removed prior to analysis.

(1) Records within ADS for which all information is an exact duplicate of another record. We remove one such record.
(2) Records within DDS that conflict with other, nearly identical DDS records. These records contain matching ID and admission date-time information with conflicting discharge date-time information. We remove 667 such records.
(3) Records for which the discharge date-time is prior to the admission date-time. We remove one such record each from ADS and DDS.
(4) Records within DDS that conflict with records in ADS. These records contain matching ID and admissions date-time information and conflicting discharge date-time information. We remove 11 such records.

### Data merging

After the data reconciliation process, we have 335,478 ADS records and 339,231 DDS records, accounting for 99.9% of the originally downloaded data. We now accept certain of these records into our final data set, attempting to match records across the two data sets when feasible. For matching, we attempt to match on three key identifiers: ID number, admission date-time, and discharge date-time.

Altogether, we accept seven different categories of records into our final data set for analysis.

(1) Records that match across ADS and DDS based on ID, admission date-time, and discharge date-time. There are 277,117 such records.
(2) Records in ADS with a missing discharge date-time that can be recovered via matching with DDS based on ID and admission date-time. There are 51,285 such records.
(3) Records in DDS that are absent from ADS because their admission date-time is prior to 2014. There are 10,723 such records.
(4) Records in DDS that are absent from ADS, but that have admission date-time 2014 or later. There are 106 such records. We presume that their absence from ADS is a clerical error.
(5) Records in ADS that are missing from DDS despite having discharge date-time information. There are 27 such records. We presume their absence from DDS is a clerical error.
(6) Records in ADS that have missing discharge date-time information and that are found in the *Current Inmates* data set. There are 4,814 such records.
(7) Records in the *Current Inmates* data set that are found neither in ADS nor DDS. There are 253 such records.

At this stage, our data set has 344,325 records. This data set has no internal conflicts on key identifiers (ID, admission date-time, and discharge date-time) and it has complete information in those variables except for cases of missing discharge date-times for currently incarcerated individuals. In Data Cleaning, below, we will remove a small number of additional records.

### Data cleaning

Though we have eliminated conflicts on key matching variables, there remain conflicts on demographic variables. For instance, there may be an individual who is designated as Black in ADS and Asian in DDS. Another individual may be listed as male for one record in ADS and may have missing gender information for another record in ADS.

We resolve race demographics in the following manner. For each unique ID number, we examine all data that we have for that individual’s race, both (potentially) across multiple incarceration episodes and (potentially) across data gleaned from ADS and DDS. In cases of disagreement, we remove all missing values and take the mode of the remaining data as the individual’s race. If there is no unique mode either because all data were missing or because there is a tie in the data, we assign NA to represent missing data. This procedure resolves race information for 13,621 records corresponding to 10,941 individuals. We perform a similar procedure for gender, which resolves 938 records for 801 individuals. It is possible that demographic discrepancies are due to changes in identity, for example, gender presentation for transgender individuals. However, because the data does not explicitly address transgender identity, we follow the procedure above. In Discussion and Conclusions, we make recommendations for improving data practices to make them more humane and to mitigate erasure of transgender individuals and other marginalized groups.

We also resolve discrepancies in status code and top charge code. Here, comparisons across records for the same individual are not relevant, as each incarceration episode may differ on these variables. However, for records that were matched across ADS and DDS, these variables should, but do not always, match. For each inconsistency, we check whether it is due to missing information or to conflicting information. For missing information, we simply accept the information that we do have. For direct conflicts, we set the final value of the variable to be NA to represent missing data. Overall, we find 21,299 explicit conflicts for status code and 3,269 for top charge code.

Next, we clean age at discharge. The distribution has a long tail of age up to 93 years old, and then a gap, with 45 individuals recorded with ages 117 or older. For these 45 records, we set age to NA. For the remaining data, there are inconsistencies in age information among many individuals with multiple incarcerations. For example, an individual discharged in 2014 may show an age of 43 at time of discharge, and may show an age of 44 for an incarceration that took place from 2019—2020. To remedy these inconsistencies, we examine all individuals with multiple incarcerations and we impose three different sets of changes.

(1) For 220,299 records comprising 63,552 individuals, there is inconsistent age data. That is to say, each of these individuals has multiple incarceration records which imply different birth dates, calculated as the difference between their discharge dates and ages at discharge. For these individuals, we calculate a birth date that minimizes the sum of the squared differences between our imputed birth date and the implied birth dates. Using this imputed birth date and the individual’s discharge dates, we calculate imputed ages at discharge. Our imputed ages differ from the original raw values by less than one year on average.
(2) For 422 records comprising 420 individuals, age data is missing. We recover it by using the same process as in (1) to compute a theoretical birth date from records that do have age data and come from the same individuals.
(3) For 13,147 records comprising 2,111 individuals, we attempt to apply the same computation described above. For each of these individuals, age adjusts by over five years for at least one record. We assume the age data is corrupt and set it to NA for all records.

We also examine the date-time of admission and date-time of discharge to create a derived variable, the duration of incarceration. For currently incarcerated individuals, we use the date-time of data acquisition instead of the date-time of discharge, since the latter does not exist. There are four cases we deal with.

(1) For 108 records, the duration of incarceration exceeds five years. Because the city corrections system is for short-term incarceration, we presume these records contain clerical errors in date-time of admission and/or discharge. Without accurate knowledge of admission/discharge information, we cannot analyze these records and so we exclude them. These eliminated 108 records would have made up less than 0.1% of the records in our final data set, and so we do not expect the elimination of these outliers to have a substantial impact on our conclusions.
(2) For 64 records, discharge occurs on the day prior to admission. We assume these are clerical errors, and we modify the discharge date-time to be 11:59 p.m. on the day of admission. For these records we count the duration of incarceration as one-half day.
(3) For 2218 records, discharge occurs before admission but on the same day. Similar to the case above, we modify the discharge time to be 11:59 and count the duration of the incarceration as one-half day.
(4) For the remaining 341,935 records, we subtract the date of discharge from the date of admission to obtain the duration of incarceration in days.

### Final data set

Our final data set is available at http://bit.ly/qside-nyc-jail-data. It comprises 344,217 incarceration records in the New York City jail system for individuals admitted and/or discharged from 2014 through 2020. We now enumerate the fields in the data set and provide some contextual and summary information about them.

ID number. As mentioned previously, ID is a unique identifier. There are 163,077 IDs in our data, indicating multiple incarcerations for some individuals. The median number of incarceration episodes is one and the mean is just over two.

Race. Perhaps surprisingly, in the ADS and DDS raw data that we download from the city’s data portal, the only values for race are Asian, Black, and Unknown, which we code as NA. The data disallows meaningful analysis of race. We discuss this issue further in Discussion and Conclusions. We find that 1.5% of incarceration episodes are known to be associated with an Asian individual and 54.0% with a Black individual. The remaining 44.5% of incarceration episodes are associated with individuals of unknown race (which could include Asian and Black).

Gender. The city codes gender as binary using the terms “female” and “male.” We find that 9.0% of incarceration episodes are associated with female individuals and 90.8% with male individuals. The remaining 0.2% of episodes have missing gender information. The median number of incarcerations is one for both males and for females, and the mean number is 1.8 for females and 2.2 for males. In Discussion and Conclusions, we make several recommendations for inclusive practices pertaining to gender data.

Age at discharge. We have age at discharge for 94.7% of records in our data set. Excluding records for ongoing incarcerations (which have no discharge date), we have age for 96.1% of our data. The mean age is 37 years old.

Admission date-time. The corrections system records the incarcerated individual’s date and time of admission. All records in our data set have an admission date-time.

Discharge date-time. The corrections system records the incarcerated individual’s date and time of discharge. For 1.5% of records in our data set, there is no discharge date-time. These records represent incarcerations that were ongoing at the time of data collection.

Duration. As mentioned, we derive this variable by taking the difference in discharge and admission dates for each episode. For the 25,522 records for which admission and discharge are on the same day, we count the duration as one-half day. Overall, the mean is 64 days, with 66 days for males and 45 days for females.

Status. Associated with each incarceration episode is a code that describes the individual’s status within the corrections system. Explanations of each status code can be found in [24].

Top charge code, description and class. For each incarceration episode, the city’s data provides the legal charge that is most responsible for the incarceration. This charge appears as an alphanumeric code that refers to a section of state law. From this charge code, we extract from the New York state laws website [25] a description of the crime and the class of the charge. For example, one top charge code in the data set is 140.20. This crime is “burglary in the third degree” and state law specifies that it is a Class D Felony.

Scrape date. We have recorded the date we downloaded raw data, namely, January 9, 2021 for all records.

It is worth contrasting our data set with another resource, the Vera Institute for Justice’s JailVizNYC [24], an online, public-facing, interactive tool for exploring New York City jail data. We conjecture that JailVizNYC is built by scraping the city’s *Daily Inmates in Custody* data set each day. As a result, this data set is richer and more granular than the one we have constructed. Unfortunately, the Vera Institute’s historical data only covers the time period June 25, 2017 through November 11, 2019, and thus cannot be used for studying incarceration during COVID. Additionally, it appears that the underlying data set is not readily available to the public. However, for our study, [24] was useful as a validation of our own data for the dates that are shared between the two.

## Analysis and results

We now use our compiled data set to examine four questions related to New York City’s discharge policies during COVID. First, we examine the historical data for the the jail population and find an unprecedented decrease during the week of March 23—29, 2020. It was at the start of this week that Mayor de Blasio made his public statement ordering the discharge of incarcerated individuals who were at a high risk of contracting COVID-19, and were at a low risk of reoffending. We then study duration of incarceration, age at discharge time, and likelihood of readmission to jail for individuals discharged during this week. For the remainder of this paper, we refer to March 23—29 (of any year) as our focus week.

**Fig 1:**
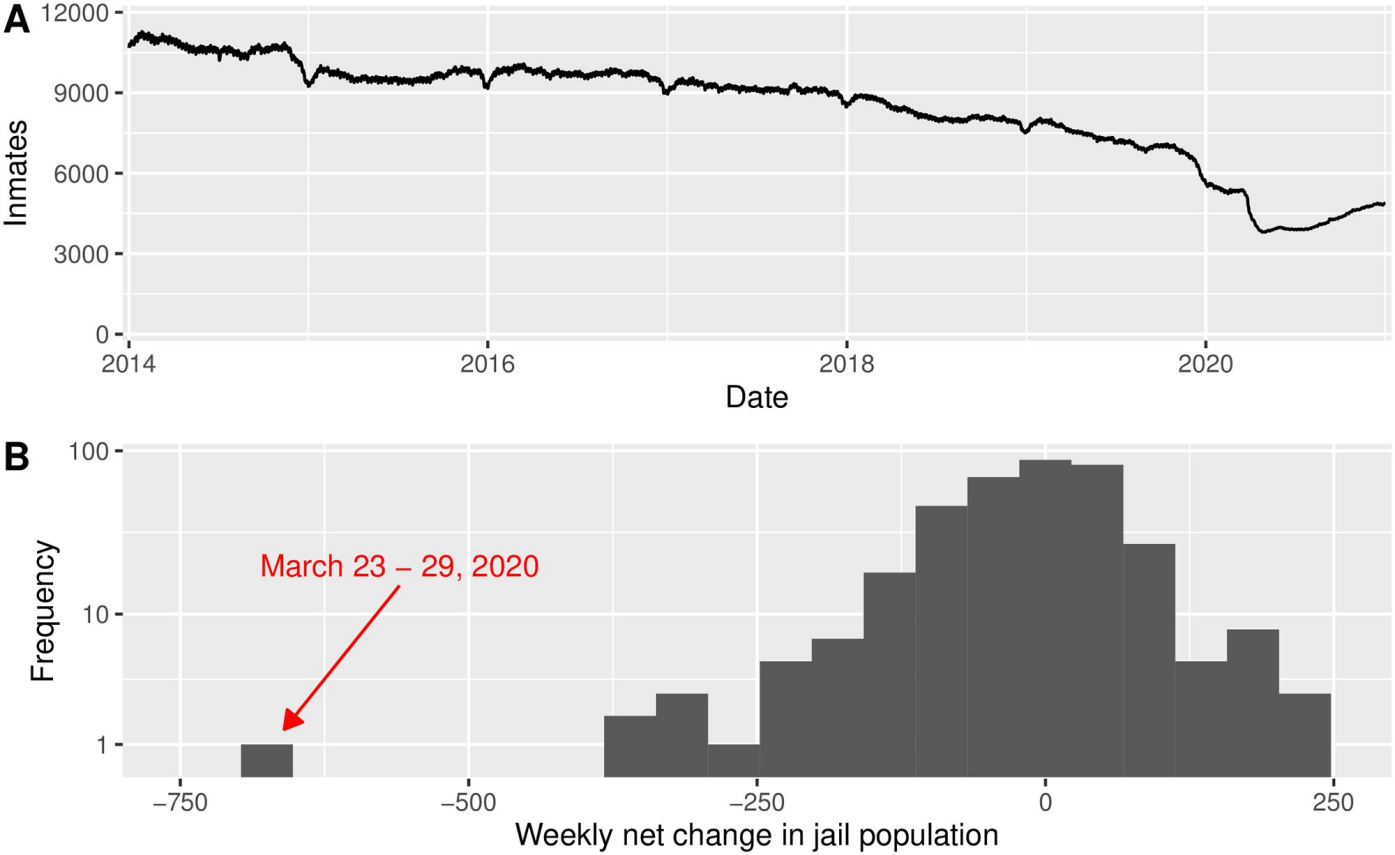
Jail population in New York City, 2014–2020. (A) Incarcerated population over time. (B) Histogram of weekly net change in jail population. The week of March 23—29, 2020 is when Mayor Bill de Blasio ordered discharge of individuals at high risk of contracting COVID-19 and at low risk of reoffending.

We begin by visualizing the historical jail population, shown in Fig 1A. Several features stand out. First, there are visible dips in the population centered around the end of calendar years. Unfortunately, the data does not provide an explanation for these dips. They could be related to decreases in crime at the ends of calendar years, decreases in policing during those same times, and/or result from a systematic data keeping error. Additionally, there is fluctuation on a weekly scale. We see local maxima in the jail population on Sundays. By examining the daily data for discharge and admission separately (rather than the total population, as shown in the figure) we find that both admissions and discharges decrease on weekends, but that discharges decrease more than admissions do, driving an overall increase in jail population.

There are two other notable features of the time series. First, in late 2019 there was a substantial decline in jail population. This decline is coincident with the start of the retroactive application of the state’s bail reform law. As discussed in Introduction, this reform led to the discharge of approximately 1,700 pretrial detainees who had been in jail precisely because they were unable to pay bail. Not long thereafter, there is an even steeper decline in jail population. As we mentioned above, this decline is during the focus week in 2020 and was catalyzed by de Blasio’s policy announcement.

To better understand the extent of this decline, we calculate the weekly net change in jail population for each weeks in our data set. Fig 1B provides a histogram of these weekly net changes. Notably, there is a single outlier: the focus week in 2020, that is, March 23—29. During this week, the jail population decreased by 662 individuals. This decrease is nearly 300 individuals larger in magnitude than the next most substantial decrease (3.3 standard deviations to the left) and is driven by both a decrease in admissions and an increase in discharges.

Because we are interested in Mayor de Blasio’s stated discharge policy, we now analyze the 819 individuals discharged during the focus week, March 23—29. To control for seasonal effects, we compare these to the 6,725 discharges in our data occurring during the focus week of all prior years, back to 2014. We will probe how certain factors in our data set are associated (or not) with discharge. Even though our data set contains information about incarceration status, we do not analyze it because of the large amount of missing data. Restricting attention to the focus week for 2014—2019, status code is missing for 5.5% of incarceration episodes, but for 2020, it is missing for 43.6%. Similarly, though we have some information about the top charge associated with each incarceration, it is missing for 67.7% of the records across the entire data set, so we do not analyze it. The factors that we are able to analyze are duration of incarceration, age at discharge time, and readmission.

### Duration of incarceration

**Fig 2:**
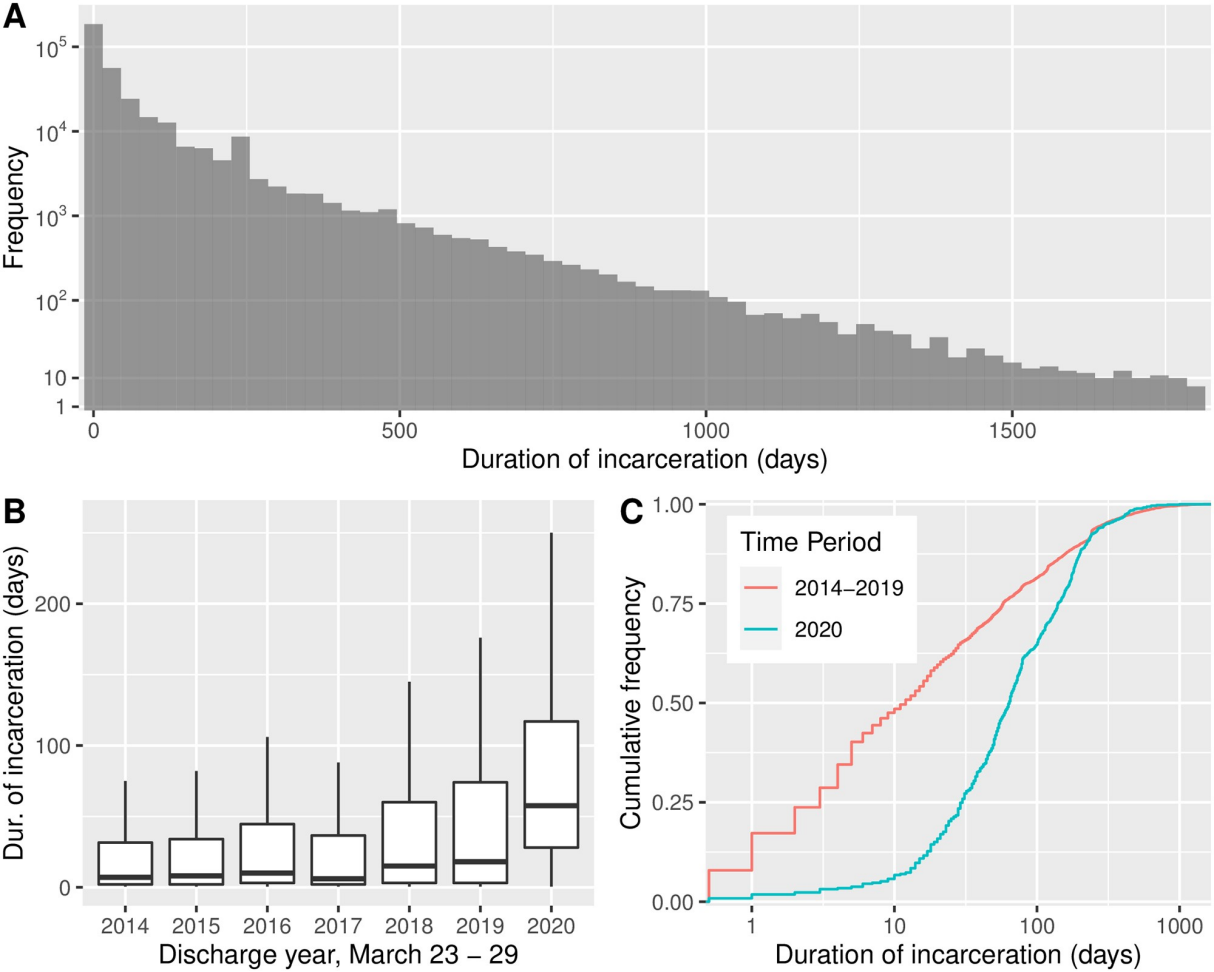
Duration of New York City incarceration episodes, 2014–2020. (A) Histogram of all episodes. Because the frequencies vary over many orders of magnitude, we use a pseudo-log scale on the vertical. (B) Distributions of duration for individuals discharged March 23—29, separated by year and shown as box plots. The distribution for 2020 appears to have a substantially higher median value. (C) Cumulative distribution function for the data in panel B, with years 2014—2019 combined. From a Kolmogorov-Smirnov test (see text) we conclude that for March 23—29 discharges, the individuals discharged in 2020 tended to have served more time than individuals in previous years.

We provide results about duration of incarceration in Fig 2. Panel A shows a histogram over the entire data set. Because the frequencies in the histogram vary over many orders of magnitude, we use a pseudo-log scale on the vertical. Panel B shows, via box plots, the distributions of duration, separated by year. The distribution for 2020 appears to have a substantially higher median value as compared to other years. To test whether duration was statistically significantly different in 2020, we perform a Kolmogorov-Smirnov (KS) test. More specifically, we test the null hypothesis that the cumulative distribution function (CDF) of durations of incarceration for individuals discharged during the focus week, 2020 does not lie below that of the CDF for those discharged during the focus week in all previous years (combined). The alternative hypothesis is that the 2020 distribution lies below the distribution for previous years. Panel C displays the two CDFs. The value of the KS test statistic is *D* = 0.98 with *p*-value *p* < 0.001. Therefore, we reject the null hypothesis and conclude that the CDF of durations for 2020 lies below and to the right of the CDF for previous years. That is to say, we conclude that for March 23—29 discharges, the individuals discharged in 2020 tended to have incarcerations of longer duration than individuals in previous years.

### Age at discharge

**Fig 3:**
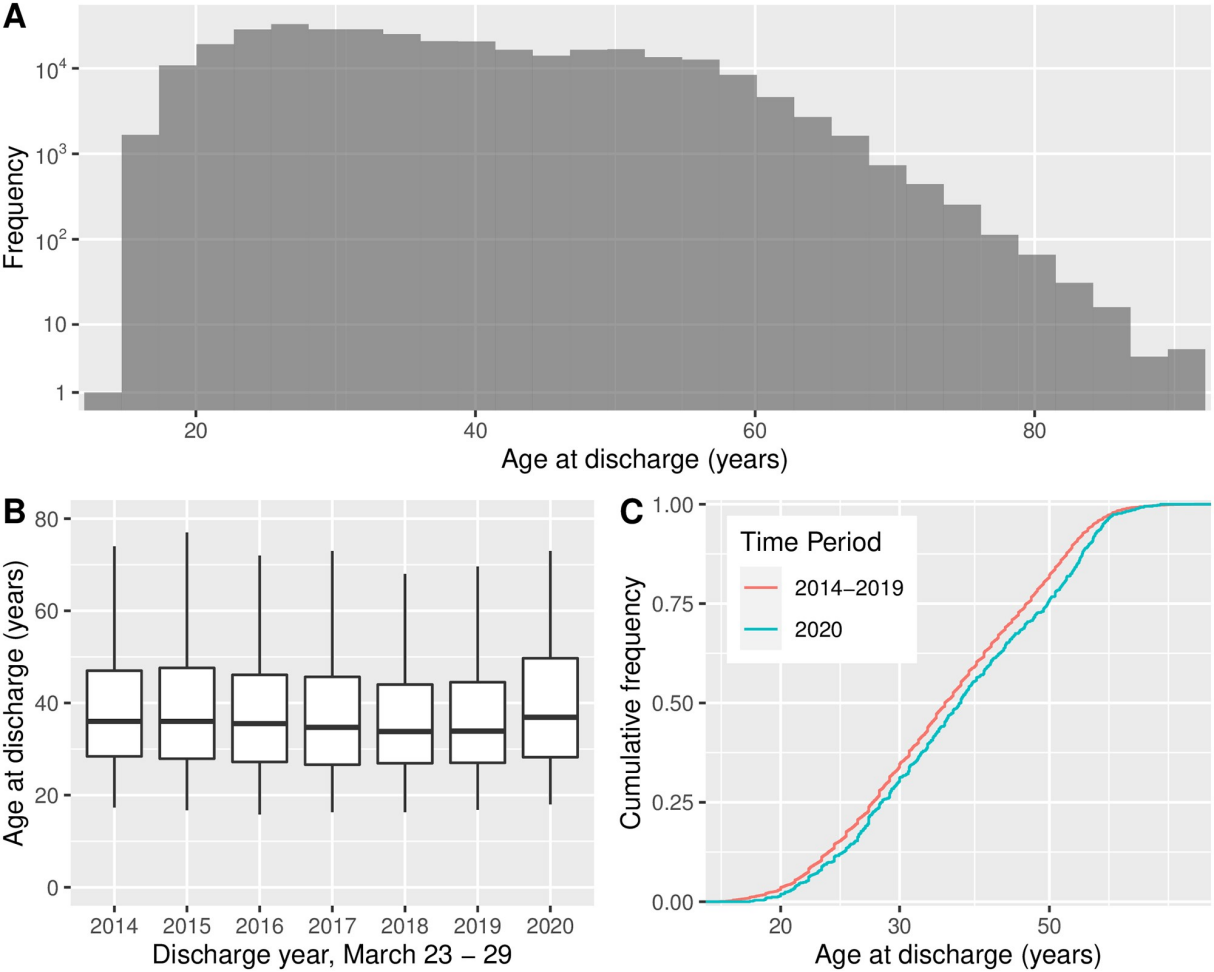
Age at discharge for New York City incarceration episodes, 2014–2020. (A) Histogram for all episodes. Because the frequencies vary over many orders of magnitude, we use a pseudo-log scale on the vertical. (B) Distributions of age for individuals discharged March 23—29, separated by year and shown as box plots. (C) Cumulative distribution function for the data in panel B, with years 2014—2019 combined. From a Kolmogorov-Smirnov test (see text) we conclude that for March 23—29 discharges, individuals discharged in 2020 tended to be older than in previous years, with a modest median diference of approximately two years.

We provide results about age at discharge in Fig 3. Panel A shows a histogram over the entire data set. Because the frequencies in the histogram vary over many orders of magnitude, we use a pseudo-log scale on the vertical. Panel B shows, via box plots, the distributions of age, separated by year. To test whether age was statistically significantly different in 2020, we perform a KS test. More specifically, we test the null hypothesis that the CDF of age of individuals discharged during the focus week, 2020 does not lie below that of the CDF for those discharged the same week in all other years (combined). Panel C displays the two CDFs. The value of the KS test statistic is *D* = 0.068 with *p*-value *p* < 0.01. Therefore, we reject the null hypothesis and conclude that the CDF of age for 2020 lies below and to the right of the CDF for previous years. That is to say, we conclude that for March 23—29 discharges, the individuals discharged in 2020 tended to be older on average. The difference is, as stated, statistically significant. The magnitude of the difference is modest, with the median value for 2020 approximately two years older than for previous years.

### Readmission

As we have mentioned, during the focus week of 2020, Mayor de Blasio ordered the release of incarcerated individuals who were at low risk of reoffending. Because there is no definitive way to know whether an individual has committed a crime, we take readmission to jail as a proxy for reoffense and study readmission within our data. Specifically, we fit a logistic regression model for a dichotomous outcome variable of whether or not a person discharged during a given calendar year was readmitted in the same calendar year.

In designing the regression, there are several important factors to keep in mind. First, the reason we use a calendar year time horizon is to make a fair comparison between years. The target of our study is March 23—29, 2020, and our data set has coverage only through the end of 2020. Second, and relatedly, individuals released later in the year would of course have less time to commit a crime and be apprehended than those released earlier in the year. To control for this effect, we add an indicator fixed effect for release during our focus week, March 23—29. Third, it is possible that the year of release could have an impact on readmission. For example, perhaps 2020 had lower readmission rates overall than other years, not just for those individuals released during our focus week. To control for this, we add an indicator fixed effect for release during 2020. Finally, our target of estimation is the effect on readmission of being released specifically during March 23—29, 2020. Thus, we also include an interaction effect between the two aforementioned fixed effects. This allows us to estimate the association between release during the focus week of 2020 and readmission, while controlling both for the marginal effects of our focus week and of 2020.

We show the results of our logistic regression estimation in Table 1. Key results are as follows. First, we observe an estimate of 0.473 associated with the focus week covariate. This positive and statistically significant effect suggests a positive association between release during March 23—29 and the log-odds of readmission in the same calendar year, controlling for all other variables in the model. Second, we observe an estimate of −0.542 associated with the 2020 covariate. This negative estimate is also statistically significant, and suggests a negative association between release during 2020 and the log-odds of readmission in the same calendar year, controlling for all other variables in the model. Lastly, our statistically significant estimate of −0.260 for the interaction effect suggests that, after controlling for the marginal effects of release during the focus week and release during 2020, release specifically during March 23—29, 2020 has a negative association with the log-odds of readmission. In other words, all else being equal, our model would predict a lower probability of readmission for an individual released during our focus week in 2020.

**Table 1:** Logistic regression model for readmission. As a proxy for reoffense, we use a dichotomous outcome variable indicating whether or not an individual discharged during a given calendar year was readmitted in the same calendar year. The model includes four parameters: an intercept; an indicator fixed effect for release during the week of March 23—29; an indicator fixed effect for release during 2020; and an interaction effect for release specifically during March 23—29, 2020.

Variable	Estimate	Standard Error
Intercept	−1.040***	0.004
Focus week	−0.473***	0.026
2020	−0.542***	0.022
Interaction of focus week & 2020	−0.260**	0.093

*** *p* < 0.001,

** *p* < 0.01,

* *p* < 0.05.

In order to assess the robustness of our conclusions, we show the results of a second logistic regression model in Table 2. In this second model, we include a fixed effect for incarceration duration. It is possible, for example, that the negative estimate of the parameter associated with focus week in the first model arises because people who are incarcerated longer are less likely to be readmitted. When we incorporate duration into our model, the estimate of the interaction effect becomes slightly less negative, increasing to −0.228, but it remains statistically significant.

**Table 2:** Logistic regression model for readmission with additional parameter. As a proxy for reoffense, we use a dichotomous outcome variable indicating whether or not an individual discharged during a given calendar year was readmitted in the same calendar year. The model includes five parameters: an intercept; an indicator fixed effect for release during the week of March 23—29; an indicator fixed effect for release during 2020; an interaction effect for release specifically during March 23—29, 2020; and a fixed effect for incarceration duration. The model is similar to that of Table 1 but adds the duration fixed effect.

Variable	Estimate	Standard Error
Intercept	−0.935***	0.004
Focus week	−0.480***	0.026
2020	−0.505***	0.022
Interaction of focus week & 2020	−0.228***	0.093
Duration	−0.002***	0.000

*** *p* < 0.001,

** *p* < 0.01,

* *p* < 0.05.

Crucially, the interpretation of these parameter estimates is not causal. That is to say, neither our model nor our conclusions suggest that release during the focus week of 2020 caused inmates to be readmitted at a lower rate. Instead, our model merely predicts a lower probability of readmission for these individuals. This lower probability could come from a number of factors, including a causal effect, exogenous factors, or randomness in the data. Nonetheless, our models do help identify associations that are present in the data.

## Policy simulation

We have shown that during the early stages of the COVID-19 pandemic, New York City chose to release incarcerated individuals, and especially the longest-serving individuals, from its jail system. With such releases now demonstrated to be feasible, and keeping in mind that the city’s jail system is intended for short-term incarceration, we now examine how the jail population would have looked over the past six years had caps in incarceration been in place. This question is related to both human rights concerns and, given the high cost of incarceration, to budgetary concerns [26].

**Fig 4:**
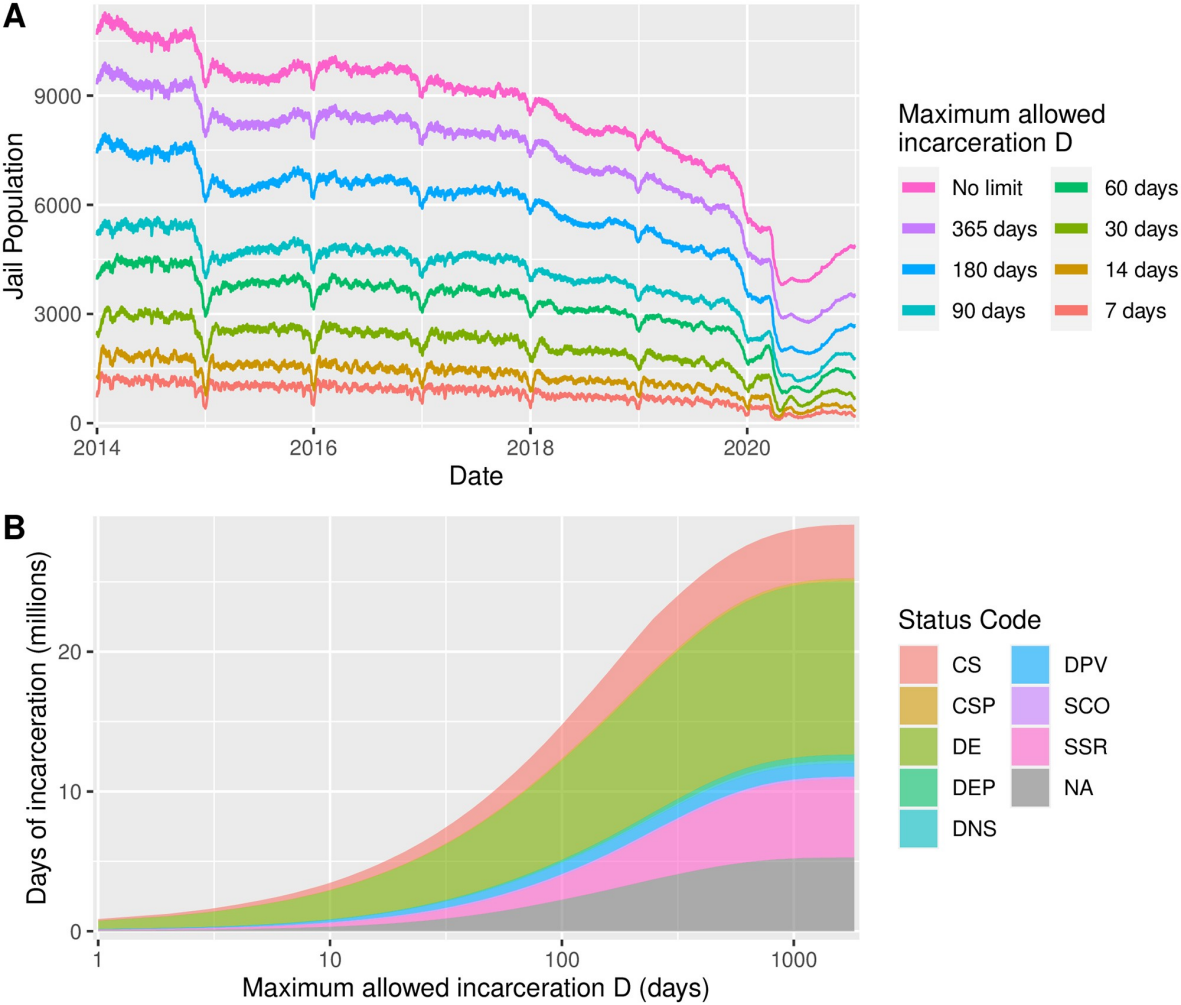
Effect on historical jail population of hypothetical incarceration caps in New York City, 2014–2020. (A) Time series of the jail population for several values of the incarceration cap *D*. The top curve (pink) represents the true historical jail population. (B) Effect of incarceration cap on the jail system as a function of *D*, which we place on a log axis. If incarcerations were limited to one year, the system would experience a 15% decrease in person-days of incarceration. With a cap of *D* = 100 days, the reduction would be over 50%. Additionally, as the cap becomes more stringent (smaller), we see a drastic reduction in the number of pre-trial detainees and a substantial proportional decrease for sentenced state-ready individuals. Status codes are as follows: CS = City Sentenced; CSP = City Sentenced with VP Warrant; DE = Detainee; DEP = Detainee Parole Violator; DNS = Detainee Newly Sentenced to State Time; DPV = Detainee Technical Parole Violator; SCO = State Prisoner Court Order; SSR = Sentenced State Ready; NA = Missing Data.

Specifically, we perform policy experiments in which we choose a maximum allowed duration of incarceration *D* days for various values of *D*. For each *D*, we find all incarceration records in our data set for which the duration of incarceration was longer than *D* days. For these records, we artificially reduce the duration of incarceration to *D* and we edit the discharge dates to be commensurate. Fig 4 summarizes our analysis of these modified data sets. Our policy simulation is carried out in an idealized setting; there are many practical considerations that would have to be addressed in institutional incarceration caps, and we mention these in Discussion and Conclusions.

Fig 4A shows time series of the jail population for several values of the incarceration cap *D*. The top curve, labeled “No limit,” is the actual jail population. The remaining curves comprise results for caps ranging from one week through one year. Because we are interested in the resources used by the jail system for long-term incarceration, panel B summarizes the effect of different incarceration caps *D* from one day through five years, broken down by status code. For each value of *D*, we construct a time series like those in panel A and add the total population each day from 2014 through 2020, yielding a total number of days of incarceration. Mathematically, this is similar to taking the integral of the time series. In terms of policy, the calculation measures the number of person-days of incarceration spent by the system. Very crudely, this count would be the same as the number of lunches served to individuals. We show the horizontal axis on a log scale so that we can consider a large range of *D*, and as a result, the curve appears sigmoidal. It is striking that if incarcerations were limited to one year, the system would experience a 15% decrease in person-days of incarceration. With a cap of *D* = 100 days, the reduction would be over 50%.

Additionally, by examining the colored areas, we can see the effect of the incarceration cap on individuals with different status codes. As the cap becomes more stringent (smaller), there is a drastic reduction in the number of pre-trial detainees (DE). This result may be of particular interest to bail reform advocates, who, as we have discussed above, have called for policies that reduce the incarceration of this group. Additionally, the greatest *proportional* decrease is for sentenced state-ready (SSR) individuals, that is, people who have been convicted of a crime and are awaiting transfer to a long-term state prison. This result suggests that expediting the transfer process may play an outsized role in reducing person-days of jail incarceration.

## Data recommendations

The accuracy of all of our conclusions is limited by the accuracy of the underlying data. New York City’s recent efforts to publicize important data on their Open Data Portal are laudable, but researchers across disciplines from public health to urban planning have noted the data sets need improvement for complete accuracy and transparency [27, 28]. We find the same is true with the jail data we use. We now highlight some challenges of the data, and ways in which New York City’s public jail data should be improved. Because we are not privy to details about the internal usage of data within city systems, we do not make recommendations for it. Though internal and external data issues are inextricably linked, we focus on aspects of the data that affect the public’s ability to understand incarceration policies and outcomes. We make the following recommendations to policymakers and data stewards within the city government.

(1) The city makes historical data available only for admission and discharge into the jail system, as opposed to a daily roster of incarcerated individuals. To know the jail population on any specific past date requires data merging and reconciliation operations that are quite involved, as we described at length in Methods.
  Recommendation: There should be a single public data set that reports incarceration records. New records should be appended and pushed to the city’s data portal every day.
(2) DDS and ADS classify race/ethnicity as either Black, Asian, or Unknown. Assuming accuracy of the data provided, this means that the only aggregate racial/ethnic demographics that can be known are lower bounds on the number of incarceration episodes for Black and Asian individuals. The picture of the jail’s historical racial/ethnic demographics is, therefore, extremely imprecise. By contrast, the *Daily Inmates in Custody* data set classifies race/ethnicity as “A,” “B,” “I,” “O,” “U,” or “W.” While no data dictionary is provided to explain the meaning of this coding, we assume that “W,” indicates white, and therefore the data would at least allow one to differentiate white individuals from others, although as mentioned above, the historical data is not archived. Overall, the aforementioned limitations preclude research into racial equity.
  Recommendation: In the data file recommended in (1), race/ethnicity should be reported as a coded categorical variable chosen by the incarcerated individual. The coding should use a rich set of categories that, while necessarily incomplete, strive to maintain dignity. One plausible set of categories might be: Asian, Black, Latinx, Middle Eastern/North African, Native American/Alaska Native, Pacific Islander/Native Hawaiian, white, An Identity Not Listed Here, and Prefer Not to Say. Individuals should be allowed to chose more than one category in order to allow for multiple identities.
(3) The current coding of gender as binary (male/female) is limiting and inhumane.
  Recommendation: Gender should be reported as a coded categorical variable chosen by the incarcerated individual. The coding should use a rich set of categories that, while necessarily incomplete, strive to maintain dignity and to better describe the full expression of human gender. In this list of categories, the terms man and woman should replace male and female, as the latter are generally used to indicate specific biological characteristics. For a jail system, gender, and not biology, is more appropriate to track. One plausible set of gender categories might be: Agender, Cisgender Man, Cisgender Woman, Nonbinary/Gender Noncomforming, Transgender Man, Transgender Woman, An Identity Not Listed Here, and Prefer Not to Say.
(4) Information about age at discharge is inconsistent, as discussed in Data Cleaning. That is to say, for many individuals with multiple incarceration episodes, the difference between discharge dates may be years off from the difference in ages at discharge. We addressed the inconsistency using a simple error-minimization procedure, but consistent age data is clearly preferable.
  Recommendation: Birth dates should be recorded in internal data, but not public-facing data. Public-facing data should provide age at discharge based on the automatically calculated difference between discharge date and birth date.
(5) The status code is missing for over 6% of incarceration episodes. The missing data precludes a public understanding of those individuals’ fate within the criminal justice system.
  Recommendation: Accurate and complete reporting of status code must be achieved for public transparency. Records with status codes missing in the public data should be flagged and remediated. The data portal should provide an accompanying glossary that explains the meaning of all status codes.
(6) The top charge responsible for each incarceration is not in a standardized form. Most charges are denoted by their section and clause in the New York State Penal Code. However, some individuals have vehicle code violations, which are sometimes (but not always) denoted with the three-letter abbreviation VTL. These charges were much less likely to have consistent entries. For example, the violation in Section 511-A of the vehicle code (facilitating aggravated unlicensed operation of a motor vehicle), was coded different ways in different records in the data, including “VTL 511,” “511(1)(A),” “511(2)(A),” “VTL 511(3),” “511(3)(A),” “511-A(3),” and “511(3)(I).” Furthermore, some top charge codes have no clear connection to any written law. For example, nearly 1000 individuals are charged with “000.00.” These sorts of data issues render it difficult or impossible for the public to know the reasons individuals are being incarcerated, thus hindering transparency.
  Recommendation: Top charge code should be standardized and made a categorical variable rather than a free-text one. The data portal should provide an accompanying glossary that explains the meaning of the codes and links to relevant sections of the law.
(7) As discussed in Data Cleaning, the raw data we downloaded contains some demonstrable and some likely errors in admission and discharge date-time. Suspect records include those with incarceration durations of many years, and those for which discharge occurs before admission. In general, these anomalies cast some doubt on the accuracy of admission and discharge date-time data. Thus, it is difficult for the public to confidently know for how long individuals are being incarcerated.
  Recommendation: It is critical to report admission and discharge date accurately. To avoid future inaccuracies in these data would likely require amending internal data-keeping and information technology procedures to which we are not privy. Nonetheless, addressing the issue should be a top-line priority.

## Discussion and conclusions

During the week of March 23—29, 2020, there was a dramatic and unprecedented reduction in the New York City jail population. This reduction was substantially larger than any other one in our data set, which dates back to 2014. Individuals discharged during this week had much longer durations of incarceration than those discharged during the same week in previous years. In concordance with de Blasio’s stated policy, we find that being discharged during the focus week is associated with a lower probability of readmission as compared to being discharged during the same calendar week in previous years. Finally, individuals discharged during this week were also slightly, although by a statistically significant margin, older.

Early in the week, Mayor Bill de Blasio told reporters that his administration would discharge individuals who were at high risk of contracting COVID-19 and at low risk of committing future offenses. It seems plausible that individuals at high risk of contracting COVID-19 would be dramatically older than the individuals discharged during the same week in previous years. Since release of dramatically older individuals was not borne out in our data, we speculate that individuals determined to have low risk of committing further crimes made up the bulk of discharges, and that these individuals had disproportionately long durations of incarceration. If this speculation is true, then the de Blasio administration may have been successful in assessing criminal risk, since being discharged during the focus week of 2020 was associated with a lower probability of readmission as compared to being discharged during the same week in previous years. While we know de Blasio’s public statements expressing a desire to release low-risk individuals, and while we have observed the statistically significant association described above, it is of course a limitation of this study that we do not have a causal view. For instance, perhaps the individuals released during the focus week were associated with a lower probability of readmission not because they were actually lower-risk, but because policing in the city was curtailed during COVID. Nonetheless, given de Blasio’s statements and the associations we have observed, we wonder if a feasible reform for the New York City jail system might indeed be to identify and discharge low-risk individuals sooner in general.

From our policy simulations, we conclude that that setting a permanent, mandatory cap on the duration of incarceration could result in a substantial, sustainable reduction in jail population. Such a cap would also lead to a dramatic reduction in person-days of incarceration, an important metric in discussions of jail operating costs, since individuals who stay in jail the longest serve a disproportionate number of the system’s total days. Finally, caps would substantially reduce the number of person-days of incarceration for pretrial detainees and the proportion of person-days for sentenced state-ready detainees.

As we discussed in our Introduction, New York put in place bail reform early in 2020, retroactive to November, 2019. As we show in Fig 1A, this reform did indeed decrease the jail population, but it plateaued around 5,500 individuals, falling short of the city’s goal of 3,300 incarcerated individuals by 2026. We conclude that the 2020 bail reform has been insufficient, on its own, to meet that goal. Our own results hint that there exist circumstances under which the city is willing to give early discharge to individuals who were sentenced, and hence not affected by the bail reform. In our simulation, we have shown that a continuation of similar early (capped) discharge policies would allow the city to meet its goal. In particular, a cap of one year (the purple curve in Fig 1A) would do so.

We do not comment on the public safety implications of instituting a cap. Criminal justice advocates [14] and law enforcement officials [29] have ongoing disagreements regarding whether or not this kind of reduction in jail population will lead to an increase in crime. We ourselves have only a limited amount of readmission data for a small subset of individuals, and so we cannot meaningfully weigh in on this debate.

Similarly, while we have demonstrated the reduction in incarceration that a cap would achieve, we cannot comment on the viability of instituting such a cap, either in New York City or elsewhere. The reason for a long incarceration depends on status code, and we will address three cases.

First, long incarcerations for city-sentenced detainees simply reflect the sentences given. From March 23—29, 2020, our data demonstrate that some of these sentences were cut short. In general, to reduce person-days of incarceration for this group would require sentencing reform.

Second, long incarcerations for sentenced state-ready detainees reflect a failure to efficiently transfer individuals to a long-term facility. To reduce person-days of incarceration for this group would likely require more resources and bureaucratic reform at city, state, and/or federal levels.

Finally, long incarcerations for pretrial detainees like Kalief Browder could be due to: unjust court rulings that refuse to dismiss a case despite the prosecution’s inability to produce evidence; bureaucratic errors; delay requests by under-resourced public lawyers; and a city-wide caseload that overwhelms an insufficient number of city courts. To shorten the duration of pre-trial detainment beyond what bail reform has achieved would require bureaucratic reform, allocation of budget to increase the court system’s capacity, and other resources and systemic changes.

Together with de Blasio’s statements, our results suggest that in a time of crisis, the city gave immediate discharge to a dramatic number of low-risk individuals. This makes us wonder why similar policies, which target individuals with especially long jail incarcerations, are not permanently in place. Additional bail reform, sentencing reform, better articulation between city jail and other justice systems, bureaucratic reform within the city’s own system, and increased budget to reduce long incarcerations among individuals who are in jail before trial are all changes that would allow the city to fulfill its commitment to reducing jail population.
